# Genome Sequence of SG33 Strain and Recombination between Wild-Type and Vaccine Myxoma Viruses

**DOI:** 10.3201/eid1704.101146

**Published:** 2011-04

**Authors:** Christelle Camus-Bouclainville, Magalie Gretillat, Robert Py, Jacqueline Gelfi, Jean-Luc Guérin, Stéphane Bertagnoli

**Affiliations:** Author affiliations: French National Institute for Agricultural Research, Toulouse, France; and Université de Toulouse, École Nationale Vétérinaire, Toulouse

**Keywords:** Viruses, myxoma virus, Poxviridae, vaccines, rabbits, genetic recombination, research

## Abstract

Myxomatosis in Europe is the result of the release of a South America strain of myxoma virus in 1952. Several attenuated strains with origins in South America or California have since been used as vaccines in the rabbit industry. We sequenced the genome of the SG33 myxoma virus vaccine strain and compared it with those of other myxoma virus strains. We show that SG33 genome carries a large deletion in its right end. Furthermore, our data strongly suggest that the virus isolate from which SG33 is derived results from an in vivo recombination between a wild-type South America (Lausanne) strain and a California MSD-derived strain. These findings raise questions about the use of insufficiently attenuated virus in vaccination.

*Myxoma virus* is a member of the family *Poxviridae* and the genus *Leporipoxvirus* (*1*). It causes a benign infection in American rabbits (*Sylvilagus* spp*.*) but is responsible for myxomatosis in the European rabbit (*Oryctolagus cuniculus*). This systemic and lethal infection is characterized by a large myxoma at the inoculation site, a leonine facies caused by edema, and numerous secondary myxomas (*2*).

Distinct myxoma virus (MYXV) strains from South America and California have been identified; virulence of California MSW strain is higher than that of South America strains in European rabbits (*3*). In contrast, the California MSD strain is reported to be less pathogenic (*3*) and has thus been used as a basis for the generation of vaccine strains on several occasions.

MYXV was introduced in France in 1952 as a means to control wild rabbit populations (*2*), and it has since spread widely throughout Europe. The strain used had been derived from a virulent South America strain (*4*) and has been called Lausanne since 1957 (*5*). Although MYXV was introduced to control wild rabbit populations, it rapidly spread to domestic rabbits, and by 1954, 30%–40% of the rabbit industry in France had been destroyed (*2*). Shope fibroma virus (SFV) was first used as a vaccine (*6**,**7*) but was only moderately effective. Limited trials were performed (*2*) by using an MSD-derived vaccine strain developed in California by Saito et al. (*8*), but this strain was later shown to cause myxomatosis symptoms in rabbits (*9**,**10*). Further attempts to attenuate the Saito strain were made (*9*). Some of the vaccine strains used throughout Europe today, such as Borghi (*11*) and MAV (*12*), are derived from the Saito strain.

In France, another attenuated vaccine was developed by Saurat et al. (*13*) in the École Nationale Vétérinaire de Toulouse virology laboratory. MYXV SG33 strain was obtained in 1977 by serial passages on a rabbit kidney cell line and chicken embryo cells at 33°C from an isolate obtained from a wild rabbit killed in the Toulouse area in 1973 (*13*). It has since been widely used as a vaccine against myxomatosis in rabbits in France and other countries in Europe.

Preliminary analyses of the SG33 genome showed a large deletion near the right end of the genome (*14**,**15*). Cavadini et al. recently performed a partial analysis of the SG33 sequence (*16*). They amplified and sequenced 200-bp to 10,000-bp fragments from 15 genomic locations, spanning 35 MYXV genes, and demonstrated that it was highly (97%–100% identity) similar to Lausanne. However, they reported somewhat lower similarities between both strains for M138L-M139R (GenBank accession no. HM104692) and M142R-M144R (GenBank accession no. HM104702) sequences, with 84% and 89% identity, respectively. They observed 100% identity between their M138L-M139R sequence and the only available MSD sequence, a partial sequence of M138L (GenBank accession no. AF030894) (*17*). We present the analysis of the genome sequence of MYXV SG33 vaccine strain, which confirms the presence of a large right-end deletion and shows evidence of a field recombination between a wild-type and a vaccine strain.

## Materials and Methods

### Cells and Viruses

Rabbit kidney cells (RK13, ATCC CCL-37) were grown in Dulbecco modified Eagle medium (GIBCO-BRL-Invitrogen, Cergy-Pontoise, France) supplemented with 10% fetal calf serum (Eurobio, Les Ulis, France). Culture medium was supplemented with 100 units/mL penicillin and 100 µg/mL streptomycin. MYXV SG33 strain was propagated in RK13 cells grown in OptiMEM (GIBCO-BRL-Invitrogen) supplemented with 2% fetal calf serum, 100 units/mL penicillin, and 100 µg/mL streptomycin.

### Genomic DNA Preparation

MYXV-infected RK13 cells were harvested and centrifuged. The cell pellet was homogenized in TL20 (20 mmol/L Tris, 150 mmol/L NaCl, 1 mmol/L EDTA; pH 8.6), disrupted in a Dounce tissue grinder, and centrifuged at 1,200 × *g* at 4°C for 10 minutes. The supernatant fluid was laid over an equal volume of a 36% sucrose cushion in TL20 and centrifuged at 200,000 × *g* for 2 hours in an SW 41 rotor at 4°C. The pellet was homogenized in TL20, laid over a 36% sucrose cushion, and recentrifuged. The new pellet was homogenized in TL20 and run into a linear 30%–65% sucrose gradient by centrifugation at 200,000 × *g* for 3 hours. The viral band was harvested and diluted in TL20 and then centrifuged at 130,000 × *g* for 1 hour. The pellet was homogenized in TL10 (1 mmol/L Tris, 150 mmol/L NaCl, 1 mmol/L EDTA; pH 8.6). After addition of 20% (vol/vol) of a 10% (wt/vol) sodium dodecyl sulfate solution, 20 µL of 10 µg/µL proteinase K of viral suspension, and 10% (vol/vol) of 20 mg/mL RNase A, the suspension was incubated at 50°C for 90 minutes with agitation. DNA was extracted by using a phenol/chloroform protocol and precipitated with 5 mol/L NaCl, 100% ethanol, rinsed with 70% ethanol, and resuspended in water.

### DNA Sequencing and Sequence Analysis

SG33 genomic DNA sequencing and assembly was performed at Beckman Coulter Genomics (Danvers, MA, USA) by using the Roche (Basel, Switzerland) 454 Life Sciences GS FLX Titanium pyrosequencing platform. The borders of the terminal inverted sequences (TIR) sequences were amplified and sequenced by using an internal primer (5′-ACGTCTACGTCCGACTGTCC-3′ for the left TIR, and 5′-AGTCGCGTGGAGAAATCAAT-3′ for the right TIR) and an external primer (5′-AATTTATAGCTCTTAAAAAAAAGTATAACC-3′) corresponding to the 30 first bp of Lausanne sequence (GenBank accession no. AF170726.2) (*18*).

Sequence genome alignments were performed by using BLAST (*19*) and DNA Strider version 1.4 (*20*). The complete SG33 sequence has been deposited in GenBank under accession no. GQ409969.

## Results

MYXV SG33 strain DNA was extracted from infected RK13 cells and sequenced. The generated contig was aligned to Lausanne strain genome sequence (GenBank accession no. AF170726.2) (*18*), for comparison. PCR amplification and sequencing of the most external 900 bp of each TIR showed 100% identity between both strains in these regions.

The genome of MYXV Lausanne strain was completely sequenced (*18*). Its 161.8 kbp encode 171 open reading frames (ORFs). Twelve of these ORFs are present in duplicate because of their localization in the TIRs of the genome. The left and right end regions of the genome (including the TIRs) mostly contain genes involved in the virulence of MYXV, whereas essential genes are found in the central part of the genome (*18*).

We determined SG33 genome to be 148,244 bp long, >13.5 kbp shorter than that of Lausanne, which is consistent with our data indicating a large deletion at the right end of the genome (*14**,**15*). A deletion spans from the second half of M151R gene to the end of M-T1 (M001R). It was confirmed by PCR amplification of the region and resequencing (data not shown). The consequences of the deletion are the absence of 13 genes and the in-frame fusion of the truncated M151R and M001R ORFs (online Appendix Table, www.cdc.gov/EID/content/17/4/633-appT.htm).

M151R encodes Serp2, a serpin that specifically binds interleukin-1β–converting enzyme (*23*) and is involved in the pathogenesis of myxomatosis (*24*). The deletion would result in the putative translation of a Serp2 protein in which its 176 last aa are missing and replaced by the 80 C-terminal aa of M-T1 protein, a CC-chemokine inhibitor (*25*). It was shown that the reactive site loop of Serp2 corresponds to its last 40 aa (*26*). It is thus unlikely that Serp2 retains its enzymatic activity. As concerns M-T1, its structure and function rely on an N terminal signal sequence and 8 conserved cysteine residues spread throughout the protein (*27*). Thus, as with Serp2, the remaining M-T1 protein is unlikely to retain any activity. Furthermore, previous experiments showed that no protein could be specifically detected by an anti-Serp2 serum in SG33-infected cells (*23*), suggesting that the fusion protein is absent or unstable. A great proportion of the genes deleted in SG33 strain remain as a single copy in the left-end TIR (M002L, M003.1L, M003.2L, M004L, M005L, M006L, M007L, M008L, M008.1L), but M152R, M153R, M154R, and M156R are missing.

M152R encodes an atypical serpin, the deletion of which triggers an attenuation of virulence in rabbit, associated with the absence of secondary myxomas (*28*). M153R codes for a factor involved in MHCI and Fas-CD95 down-regulation. Its deletion induces a reduction of clinical signs and virulence in rabbits (*29*). M154R codes for a protein presenting 50% identity with M2L, a vaccinia virus gene that was shown to inhibit induction of NF-κB activation through an ERK2 pathway in virus-infected human embryonic kidney cells (*30*). Finally, M156R-encoded protein is a structural mimic of eukaryotic translation initiation factor eIF2α (*31*). Hence, all these genes seem to be involved in the virulence of MYXV, thus accounting for their combined deletion leading to a high attenuation of the strain.

Apart from this large deletion, SG33 genome presents other differences with Lausanne genome. Mutations in intergenic sequences are not discussed here. In contrast, we established a gene-by-gene comparison of Lausanne and SG33 strains. The genes presenting amino acid discrepancies are listed in the online Appendix Table.

Some of the differences observed with the genomic sequence of the Lausanne strain (online Appendix Table, M020L and M069L) have already been reported for other strains and were attributed to errors in the Lausanne genome sequence (*21**,**32*). They will not be further discussed.

M011L-encoded protein is involved in the regulation of apoptosis and is directed specifically to mitochondria by a short COOH-terminal region (*33*). In the SG33 genome, a substitution in M011L sequence leads to a non-sense codon and to the generation of 2 ORFs of 33 and 115 codons, respectively. The sequence surrounding the first AUG is 5′-UCGUCGAUGG-3′, which is partially divergent from KOZAK consensus (5′-gccRccAUGG-3′) (*34*) and thus consistent with the translation of the second ORF of the mRNA. The resulting protein should still have the ability to distribute in the mitochondria, because the targeting region is at the C-terminus of M11L (*33*). However, whether 1 or both of these polypeptides are actually expressed and functional remains to be clarified.

Among the other genes with major differences with regard to Lausanne, M077L is putatively lengthened by 23 N terminal amino acids because of the mutation of a stop codon upstream from the ATG (Table). Nevertheless, this potential additional coding sequence corresponds to the promoting region of M077L and might thus not be transcribed, let alone translated.

**Table:** Myxoma virus genes with amino acids discrepancies between Lausanne and SG33 sequences*

ORF	Position in genome†		Nucleotide changes or % identity‡	Amino acid changes or % identity§
	Lausanne (*18*)	SG33		
M005L	6383–4935	6369–4921	A6351G	Silent
			C6286	R33Q
M006L	7948–6422	7934–6408	G6683A	Silent
			T6608G	E447D
M011L¶	14125–13628			
M011bL		14110–14012		Initiates at M52
			A13890G	V28A
			A13857C	V39G
M011aL		14569–14126	G14103A	A8V
			G14024T	C34Stop
M020L#	20531–19197	20518–19181	20379 GAG insertion	Addition of L (aa 52)
M030L	30037–29372	30024–29359	T30011C	T10A
M031R	30138–31316	30125–31303	C30614T	A159V
M034L	36864–33847	36851–33834	T36186C	Y227C
M044R	44157–46190	44144–46177	A44593T	T146S
			A44596G	N147D
			G44940T	K261N
M047R	48288–48962	48275–48949	A48780G	T164A
M049R	49312–50604	49299–50591	G49777A	M155I
M053R	52380–53159	52367–53146	G53113A	D245N
M054R	53183–54178	53170–54165	97%	7 substitutions, 97%
M058R	56201–56953	56188–56940	C56404T	A68V
M062R	58406–58879	58393–58866	T58642C	I79T
M064R	59631–60239	59617–60222	60131 AGA insertion	Addition of E (aa 163)
M069L#	66614–66081	66598–66083	66101 T deletion	6-aa addition
M073R	70698–71279	70682–71263	C70861T	A55V
M076R	72702–75206	72686–75190	100% (72686–73782) 95% (73783–75190)	11 substitutions, 98%
M077L	75602–75174	75655–75158	91%	8 substitutions, 94% identity
			C75619A T75620C	Stop → C upstream of ATG, potential N-terminal 23 aa addition**
M078R	75608–76327	75592–76311	94%	11 substitutions, 95%
M079R	76327–76980	76311–76964	96%	4 substitutions, 98%
M080R	77017–79374	77001–79358	95% (77001–77639) 99% (77640–79361)	6 substitutions, 99%
M083L	82636–81779	82605–81763	81958–81972 deletion	218 YNVKA 222 deletion
M085R	83302–84078	83271–84047	C83976T	A225V
M092L	91923–89965	91892–89934	A90679C	S416A
M095L	94089–92971	94058–92940	A93326C A93328G	S255P
M096L	96252–94120	96221–94089	C95947T	A103T
M099L	100099–97397	100068–97366	A98212G	I630T
M111R	106301–107593	106270–107562	T107143G	V281G
M134R	125694–131693	125663–131662	G130985A	S1773N
M135R	131699–132232	131668–132201	96%	8 substitutions in second half of protein
M136R	132368–132904	132387–132929	87%	KL insertion, 26 substitutions 83%
M137R	132908–133837	132933–133862	85%	48 substitutions, 84%
M138L	134746–133877	134767–133898	84%	53 substitutions, 81%††
M139R	134806–135369	134818–135381	91%	13 substitutions, 93%
M140R	135375–137033	135387–137045	90%	51 substitutions, 90%
M141R	137069–137722	137089–137757	80%	53 substitutions, 9 insertions/ deletions, 76%
M142R	137731–138648	137768–138697	89%	A306NITRI (C-terminal) 21 substitutions, 93%
M143R	138665–139366	138701–139402	90%	13 substitutions, 94%
M144R	139411–140310	139452–140345	84%	67 substitutions, EY deletion 77%
M146R	140335–140658	140372–140695	86%	15 substitutions, 85%
M147R	140700–141563	140749–141609	84%	31 substitutions, 89%
M148R	141626–143650	141678–143799	75%	217 substitutions, 67%
M149R	143655–145124	143704–145173	85%	63 substitutions, 87% ‡‡
**M150R**	**145191–146672**	**145241–146713**	**83%**	**96 substitutions, 80% ‡‡**
**M151R**	**146684–147682**		**85% identity on 467 bp**	**84% identity on aa 1–157, C-terminal 176-aa deletion‡‡**
**M001R**	**160190–160969**		**83% id on 244 bp**	**87% identity on aa 181–260, N-terminal 180-aa deletion**
**M151R-M001R§§**		**146732–147439**		

*Boldface indicates genes at the border of SG33 deletion. ORF, open reading frame. †Stop codon not included. ‡Nucleotide changes with position in Lausanne genome, or identity percentage if too many. §Amino acid changes with position in Lausanne ORF, or no. changes and identity percentage if too many. ¶ Apparition of a stop codon, leading to the potential translation of 2 polypeptides (M011aL and M011bL) from SG33 transcript. # Identical to 6918 strain (*21*). ** Addition not probable in view of promoting region position. ††100% identity to 1,189 bp of MSD strain (*17*). ‡‡99.6% identity on 1,737 bp with MSW strain (discontinued sequences) (*22*). §§ Fusion of 2 partial ORFs as a result of deletion.

From a global point of view, when compared with the Lausanne genome, SG33 DNA exhibits a high degree of nucleotide similarity from M000.5L to half of M135R and at the end of the right TIR (M000.5R). In these regions, 108 genes encode proteins 100% identical to their Lausanne counterparts. Among these, 91 genes have nucleotide sequences that are 100% identical. In contrast, in the same regions, only 5 complete or partial ORFs (spanning from the second half of M076R to the first third of M080R) are <97% identical to their Lausanne counterparts (online Appendix Table; Figure).

From M135R to M001R, and not taking the deletion into account, identity dropped to 75%–91% (online Appendix Table; Figure). We then compared SG33 sequence with the available partial sequences of California MYXV strains MSD and MSW. As described (*16*), SG33 is 100% identical to the only MSD sequence in GenBank, a partial sequence of M138L (GenBank accession no. AF030894) (*17*). Labudovic et al. (*22*) partially sequenced MSW strain using cloned *Eco*RI and *Sal*I fragments (GenBank accession nos. CC783373–CC783446 and CC799152–CC799159). The major difference between MSW and Lausanne strain is a duplication in the left TIR of 5 complete (M151R, M152R, M153R, M154R, and M156R) and 1 partial (M150R) ORFs from the right end of the genome, causing the partial deletion of M009L (*22*). As with Lausanne, comparison between SG33 and MSW sequences clearly shows 2 different regions in SG33 sequence (Figure). From M002L to M134R, nucleotide identity between SG33 and MSW ranges from 70.9% to 95.2%, as is the case between Lausanne and MSW sequences (*22*). SG33 M076R and M080R, which were shown to be the more divergent from Lausanne in this region, showed identity within the same range (92.7% and 95.2% identity to MSW, respectively) and are thus not closer to MSW than to Lausanne. In contrast, SG33 and the available MSW sequences from M149R to M151R share 99.4% to 99.9% identity and present only 7 differences.

**Figure:**
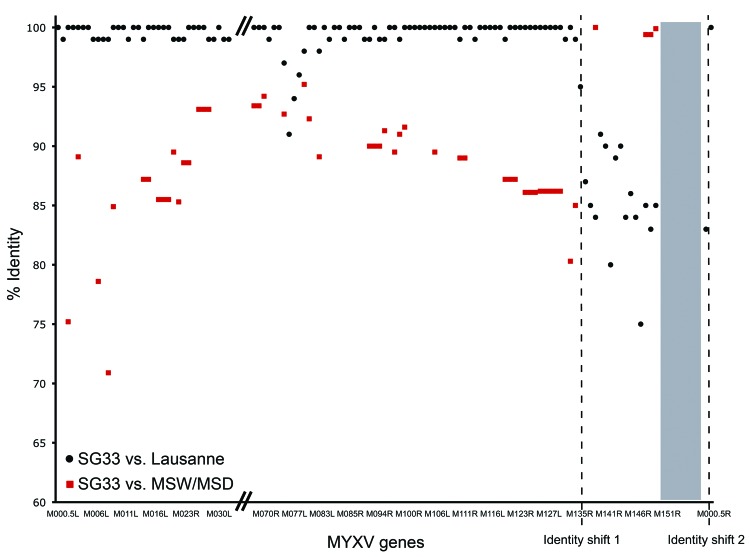
Schematic comparison of SG33 nucleic acid similarities with Lausanne and California MSD/MSW myxoma virus (MYXV) strains. Nucleotide identities were calculated between SG33 and Lausanne open reading frames and between MSW available sequences and the corresponding SG33 sequences. Dotted lines, SG33 vs. Lausanne and MSD/MSW identity shifts. Gray box, SG33 deletion.

## Discussion

Analysis of SG33 genome sequence confirmed a 13.5-kbp deletion at the right end of the genome. Notably, only 1,045 bp of the right RIT remain in SG33 sequence. This finding suggests that such a short residual sequence is sufficient for a correct genome replication. In addition, comparison with databanks showed that SG33 seems to be a composite virus, resulting from a recombination between South America (Lausanne) and California (MSW/MSD) strains. The strain from which SG33 is derived was obtained from a rabbit killed in the Toulouse area in 1973. This isolate was injected into 2 rabbits in whom classical yet delayed myxomatosis developed. One rabbit survived, and the other died 34 days after infection, which indicates that this virus was attenuated to some degree, although the number of rabbits tested is not statistically relevant. Then, serial passages on a rabbit kidney cell line and chicken embryo cells at 33°C led to the strain named SG33 (*13*).

Although somewhat attenuated, the initial viral isolate had retained enough virulence to kill rabbits. Because several genes deleted in SG33 play a critical role in virus pathogenicity, it is unlikely that this deletion was present in the initial viral isolate and is more likely the result of in vitro adaptation. It is unfortunate that this initial viral isolate was lost (R. Py and J. Gelfi, unpub. data) because sequencing of this virus would be the only way to reach a definite conclusion on this point.

However, the question of the recombination remains open. No California strain has ever been handled in the virology laboratory of École Nationale Vétérinaire de Toulouse, where SG33 was obtained (R. Py, unpub. data). In contrast, before 1970, MSD-derived Saito strain (*8**,**35*) was used for some time as a vaccine in the rabbit industry in France (*2*). It has since been demonstrated that this strain is not completely attenuated and is responsible for myxomatosis symptoms in the rabbit (*9**,**10*). It is thus possible to assume that it could disseminate and infect wild rabbits.

During the same period, Lausanne-like strains were circulating in wild rabbits in the Toulouse area. For example, the Toulouse-1 strain, which was isolated from an infected rabbit in 1952 and deposited at the Collection Nationale de Cultures de Microorganismes at Pasteur Institute (CNCM I-1592), is close to Lausanne. M151R, M152R, and M153R are 99%–100% identical to their Lausanne counterparts (*26**,**28**,**29*). Thus, the most plausible explanation of the dual origin of SG33 is that the isolate used to generate it was itself the product of a field recombination between a virulent South America strain and a vaccine California strain. The fact that the only MSD sequence available shows 100% identity with the corresponding SG33 sequence (*16*; this work), strongly supports this hypothesis.

Other occurrences of recombination of poxvirus strains have been described. It was established that malignant rabbit virus is the result of a recombination between MYXV and SFV (*36**,**37*). Nevertheless, because it was isolated from tumors induced by an uncloned stock of SFV (*38*), the recombination event most likely happened in vitro. Similarly, Gershon et al. described genetic recombination between capripoxviruses during natural transmission of wild-type strains (*39*). However, SG33 sequence might be evidence of a recombination between vaccine and virulent poxvirus strains in the field.

These findings raise the issue of the use of insufficiently attenuated live viruses, especially when used as recombinant vaccines. It was shown that loss of transgene could occur in recombinant viruses obtained from in vitro co-infection of permissive cells with a live modified vaccinia Ankara–vectored influenza vaccine and a naturally occurring cowpox virus (*40*). As previously described, the MYXV Saito strain used before 1970 was not sufficiently attenuated and was thus potentially able to disseminate and recombine with circulating wild-type strains.

Since then, SG33 and Borghi vaccine strains have been widely used in France and in Europe, and no event of virulence recovery was ever reported, which suggests that the attenuation of these strains is stable. Nevertheless, a complete sequencing of MSD strain would give clearer insight into the origin of MYXV strains now used. However, this raises the need for better knowledge of the strains used to engineer recombinant viruses, particularly at a time when poxvirus-vectored vaccines against infectious diseases and cancer are being developed.

## References

[1] Fenner F. Portraits of viruses: the poxviruses. Intervirology. 1979;11:137–57. 10.1159/000149027 372132

[2] Fenner F, Fantini B. Biological control of vertebrate pests. The history of myxomatosis—an experiment in evolution. Wallingford-Oxon (UK): CABI Publishing; 1999.

[3] Silvers L, Inglis B, Labudovic A, Janssens PA, van Leeuwen BH, Kerr PJ. Virulence and pathogenesis of the MSW and MSD strains of Californian myxoma virus in European rabbits with genetic resistance to myxomatosis compared to rabbits with no genetic resistance. Virology. 2006;348:72–83. 10.1016/j.virol.2005.12.007 16442580

[4] Bouvier G. Quelques remarques sur la myxomatose. Bull Off Int Epizoot. 1954;46:76–7.

[5] Fenner F, Marshall I. A comparison of the virulence for European rabbits (*Oryctolagus cuniculus*) of strains of myxoma virus recovered in the field in Australia, Europe and America. J Hyg (Lond). 1957;55:149–91. 10.1017/S0022172400037098 13439170 PMC2217926

[6] Shope RE. A transmissible tumor-like condition in rabbits. J Exp Med. 1932;56:793–802. 10.1084/jem.56.6.793 19870103 PMC2132208

[7] Shope RE. Infectious fibroma of rabbits: III. The serial transmission of virus myxomatosum in cottontail rabbits, and cross-immunity tests with the fibroma virus. J Exp Med. 1936;63:33–41. 10.1084/jem.63.1.33 19870458 PMC2133319

[8] Saito JK, McKercher DG, Castrucci G. Attenuation of the myxoma virus and use of the living attenuated virus as an immunizing agent for myxomatosis. J Infect Dis. 1964;114:417–28. 10.1093/infdis/114.5.417 14233132

[9] Jiran E, Sladká M, Kunstýr I. Myxomatosis of rabbits–study of virus modification. Zentralbl Veterinarmed B. 1970;17:418–28. 10.1111/j.1439-0450.1970.tb01454.x 5516854

[10] Jacotot H, Virat B, Reculard P, Vallée A, Le Bouquin MJ, Boutry JM, Study of an attenuated strain of infectious myxoma virus obtained by passage in cell cultures (MacKercher and Saito, 1964) [in French] [**PMID 6055855**]. Ann Inst Pasteur (Paris). 1967;113:221–37. 6055855

[11] Cancellotti F. Caratteristiche dello stipite vaccinale Borghi. Rivista di Coniglicoltura. 1985;3:24–31.

[12] Górski J, Mizak B, Chrobocińska M. Control of rabbit myxomatosis in Poland. Rev Sci Tech. 1994;13:869–79. 7949359 10.20506/rst.13.3.803

[13] Saurat P, Gilbert Y, Gagnière J. Study of a modified myxoma virus strain [in French]. Rev Med Vet (Toulouse). 1978;129:415–51.

[14] Guérin J, Petit F, Van Es A, Gelfi J, Py R, Bertagnoli S, Molecular analysis of myxomatosis vaccine strains SG33 and Poxlap: prophylactic and epidemiological implications [in French]. Dans: 7èmes journées de la Recherche Cunicole française. Lyon: 1998. p. 53–56.

[15] Petit F, Boucraut-Baralon C, Py R, Bertagnoli S. Analysis of myxoma virus genome using pulsed-field gel electrophoresis. Vet Microbiol. 1996;50(1–296405880):27–32. 10.1016/0378-1135(96)00014-4 8810005

[16] Cavadini P, Botti G, Barbieri I, Lavazza A, Capucci L. Molecular characterization of SG33 and Borghi vaccines used against myxomatosis. Vaccine. 2010;28:5414–20. 10.1016/j.vaccine.2010.06.017 20598407

[17] Jackson RJ, Hall D, Kerr P. Myxoma virus encodes an alpha2,3-sialyltransferase that enhances virulence. J Virol. 1999;73:2376–84. 9971821 10.1128/jvi.73.3.2376-2384.1999 PMC104483

[18] Cameron C, Hota-Mitchell S, Chen L, Barrett J, Cao JX, Macaulay C, The complete DNA sequence of myxoma virus. Virology. 1999;264:298–318. 10.1006/viro.1999.0001 10562494

[19] Altschul SF, Gish W, Miller W, Myers EW, Lipman DJ. Basic local alignment search tool. J Mol Biol. 1990;215:403–10. 2231712 10.1016/S0022-2836(05)80360-2

[20] Marck C. ‘DNA Strider’: a ‘C’ program for the fast analysis of DNA and protein sequences on the Apple Macintosh family of computers. Nucleic Acids Res. 1988;16:1829–36. 10.1093/nar/16.5.1829 2832831 PMC338177

[21] Morales M, Ramírez MA, Cano MJ, Párraga M, Castilla J, Pérez-Ordoyo LI, Genome comparison of a nonpathogenic myxoma virus field strain with its ancestor, the virulent Lausanne strain. J Virol. 2009;83:2397–403. 10.1128/JVI.02189-08 19091868 PMC2643724

[22] Labudovic A, Perkins H, van Leeuwen B, Kerr P. Sequence mapping of the Californian MSW strain of myxoma virus. Arch Virol. 2004;149:553–70. 10.1007/s00705-003-0222-6 14991443

[23] Petit F, Bertagnoli S, Gelfi J, Fassy F, Boucraut-Baralon C, Milon A. Characterization of a myxoma virus–encoded serpin-like protein with activity against interleukin-1 beta-converting enzyme. J Virol. 1996;70:5860–6. 8709205 10.1128/jvi.70.9.5860-5866.1996 PMC190603

[24] Messud-Petit F, Gelfi J, Delverdier M, Amardeilh MF, Py R, Sutter G, Serp2, an inhibitor of the interleukin-1beta–converting enzyme, is critical in the pathobiology of myxoma virus. J Virol. 1998;72:7830–9. 9733819 10.1128/jvi.72.10.7830-7839.1998 PMC110100

[25] Lalani AS, Masters J, Graham K, Liu L, Lucas A, McFadden G. Role of the myxoma virus soluble CC-chemokine inhibitor glycoprotein, M-T1, during myxoma virus pathogenesis. Virology. 1999;256:233–45. 10.1006/viro.1999.9617 10191189

[26] Turner PC, Sancho M, Thoennes S, Caputo A, Bleackley R, Moyer R. Myxoma virus Serp2 is a weak inhibitor of granzyme B and interleukin-1 beta-converting enzyme in vitro and unlike CrmA cannot block apoptosis in cowpox virus–infected cells. J Virol. 1999;73:6394–404. 10400732 10.1128/jvi.73.8.6394-6404.1999 PMC112719

[27] Graham KA, Lalani A, Macen J, Ness T, Barry M, Liu L, The T1/35kDa family of poxvirus-secreted proteins bind chemokines and modulate leukocyte influx into virus-infected tissues. Virology. 1997;229:12–24. 10.1006/viro.1996.8423 9123853

[28] Guerin JL, Gelfi J, Camus C, Delverdier M, Whisstock JC, Amardeihl MF, Characterization and functional analysis of Serp3: a novel myxoma virus–encoded serpin involved in virulence. J Gen Virol. 2001;82:1407–17. 11369885 10.1099/0022-1317-82-6-1407

[29] Guerin JL, Gelfi J, Boullier S, Delverdier M, Bellanger FA, Bertagnoli S, Myxoma virus leukemia-associated protein is responsible for major histocompatibility complex class I and Fas-CD95 down-regulation and defines scrapins, a new group of surface cellular receptor abductor proteins. J Virol. 2002;76:2912–23. 10.1128/JVI.76.6.2912-2923.2002 11861858 PMC135958

[30] Gedey R, Jin X, Hinthong O, Shisler JL. Poxviral regulation of the host NF-κB response: the vaccinia virus M2L protein inhibits induction of NF-κB activation via an ERK2 pathway in virus-infected human embryonic kidney cells. J Virol. 2006;80:8676–85. 10.1128/JVI.00935-06 16912315 PMC1563854

[31] Ramelot TA, Cort JR, Yee AA, Liu F, Goshe MB, Edwards AM, Myxoma virus immunomodulatory protein M156R is a structural mimic of eukaryotic translation initiation factor eIF2alpha. J Mol Biol. 2002;322:943–54. 10.1016/S0022-2836(02)00858-6 12367520

[32] Mossman K, Ostergaard H, Upton C, McFadden G. Myxoma virus and Shope fibroma virus encode dual-specificity tyrosine/serine phosphatases which are essential for virus viability. Virology. 1995;206:572–82. 10.1016/S0042-6822(95)80074-3 7831813

[33] Everett H, Barry M, Lee SF, Sun X, Graham K, Stone J, M11L: a novel mitochondria-localized protein of myxoma virus that blocks apoptosis of infected leukocytes. J Exp Med. 2000;191:1487–98. 10.1084/jem.191.9.1487 10790424 PMC2213443

[34] Kozak M. An analysis of 5′-noncoding sequences from 699 vertebrate messenger RNAs. Nucleic Acids Res. 1987;15:8125–48. 10.1093/nar/15.20.8125 3313277 PMC306349

[35] McKercher DG, Saito JK. An attenuated live virus vaccine for myxomatosis. Nature. 1964;202:933–4. 10.1038/202933a0 14190113

[36] Block W, Upton C, McFadden G. Tumorigenic poxviruses: genomic organization of malignant rabbit virus, a recombinant between Shope fibroma virus and myxoma virus. Virology. 1985;140:113–24. 10.1016/0042-6822(85)90450-7 2981446

[37] Upton C, Macen JL, Maranchuk RA, DeLange AM, McFadden G. Tumorigenic poxviruses: fine analysis of the recombination junctions in malignant rabbit fibroma virus, a recombinant between Shope fibroma virus and myxoma virus. Virology. 1988;166:229–39. 10.1016/0042-6822(88)90164-X 2842947

[38] Strayer DS, Cabirac G, Sell S, Leibowitz JL. Malignant rabbit fibroma virus: observations on the culture and histopathologic characteristics of a new virus-induced rabbit tumor. J Natl Cancer Inst. 1983;71:91–104. 6306326

[39] Gershon PD, Kitching RP, Hammond JM, Black DN. Poxvirus genetic recombination during natural virus transmission. J Gen Virol. 1989;70:485–9. 10.1099/0022-1317-70-2-485 2543750

[40] Hansen H, Okeke MI, Nilssen O, Traavik T. Recombinant viruses obtained from co-infection in vitro with a live vaccinia-vectored influenza vaccine and a naturally occurring cowpox virus display different plaque phenotypes and loss of the transgene. Vaccine. 2004;23:499–506. 10.1016/j.vaccine.2004.06.032 15530698

